# MANAGEMENT OF ENDOCRINE DISEASE: Postsurgical hypoparathyroidism: current treatments and future prospects for parathyroid allotransplantation

**DOI:** 10.1530/EJE-20-1367

**Published:** 2021-02-17

**Authors:** Radu Mihai, Rajesh V Thakker

**Affiliations:** 1 Department of Endocrine Surgery, Churchill Cancer Centre, Oxford University Hospitals NHS Foundation Trust, Oxford, Oxfordshire, UK; 2 Academic Endocrine Unit, Radcliffe Department of Medicine, University of Oxford, Oxford, UK

## Abstract

**Background:**

Permanent postsurgical hypoparathyroidism (POSH) is a major complication of anterior neck surgery in general and of thyroid surgery in particular. Depending on diagnostic criteria, up to 10% of patients undergoing bilateral thyroid surgery develop POSH. This leads to a multitude of symptoms that decrease the quality of life and burden the healthcare provision through complex needs for medication and treatment of specific complications, such as seizures and laryngospasm.

**Methods:**

Narrative review of current medical treatments for POSH and of the experience accumulated with parathyroid allotransplantation.

**Results:**

In most patients, POSH is controlled with regular use of calcium supplements and active vitamin D analogues but a significant proportion of patients continue to experience severe symptoms requiring repeated emergency admissions. Replacement therapy with synthetic PTH compounds (PTH1-34, Natpara® and PTH1-84, teriparatide, Forsteo®) has been assessed in multicentre trials, but the use of this medication is restricted by costs and concerns related to the risk of development of osteosarcoma. Based on recent case reports of successful allotransplantation of parathyroid tissue between siblings, there is renewed interest in this technique. Data on selection of donors, parathyroid cell preparation before allotransplantation, site and timing of transplantation, need for immunosuppression and long-term outcomes are reviewed.

**Conclusion:**

A prospective trial to assess the efficacy of parathyroid allotransplantation in patients with severely symptomatic protracted post-surgical hypoparathyroidism is warranted.

## Introduction

When Albert, a 34 years old man with multiple recent fractures, was admitted under the care of Dr Felix Mandle in Vienna in early 1920s, the initial working diagnosis was an insufficiency of the parathyroid gland function. A victim of a tram accident was used as donor, and Albert received the first recorded allotransplant of parathyroid glands. When it became apparent that this procedure had no clinical impact, Dr Mandle decided to explore Albert’s neck and this first successful parathyroidectomy started the history of parathyroid surgery (1). Currently, parathyroid surgery for primary hyperparathyroidism is one of the most common endocrine operations. It could be that in a further decade parathyroid allotransplantation (PTalloT) will become part of the surgical practice.

This paper is a narrative review of current therapeutic options for hypoparathyroidism and aims to create the background for a potential clinical trial exploring the role of allotransplantation in patients with severely symptomatic refractory hypoparathyroidism.

## Causes of postsurgical hypoparathyroidism (hypoPT)

Hypoparathyroidism (hypoPT) is defined as low serum calcium levels in the presence of inappropriately low/absent PTH levels. The condition is divided into primary hypoPT due to intrinsic genetic and autoimmune defects within the parathyroid glands, and the much more common secondary/acquired hypoPT due to impaired parathyroid function.

The vast majority of cases of secondary hypoPT are represented by postsurgical hypoPT (POSH), caused by intraoperative injury to the parathyroid glands through compromising vascular supply, thermic injury or inadvertent excision. It is rare to encounter patients with secondary hypoPT caused by rare infiltrative disorders (e.g. iron/copper overload) or by ionising radiation exposure (2).

Bilateral thyroid surgery is the cause of POSH in over 80% of patients. About 10–15% of POSH cases occur after parathyroid surgery. Radical resection of laryngeal tumours is a rare cause of POSH (under 5% of cases). The risk of POSH after thyroid surgery is influenced by several operative factors. High surgical volume (3, 4), routine parathyroid identification and *in situ* preservation of parathyroid glands (5) are all reducing the risk of POSH. In contrast, longer operations, reoperation for bleeding, the need for associated central compartment lymph node dissection and the routine use of parathyroid autotransplantation (6) increase the risk of POSH. It is expected that the intraoperative use of (auto)fluorescence for identification of parathyroid glands and for assessment of their vascular supply can reduce the risk of POSH (7).

## Epidemiology of postsurgical hypoPT

The reported incidence of POSH varies widely because different definitions are used (e.g. calcium levels under the lower limit of the normal range, or symptomatic hypocalcaemia, or low PTH levels within 4 h or within 24 h after the operation). Similarly, permanent hypoPT is diagnosed after full biochemical reassessment at 6 or 12 months after the operation, and patients who take calcium/vitamin D supplements for other indications (e.g. osteoporosis) are not always excluded in large population studies.

Transient POSH is estimated to occur in 25–80% of patients after neck surgery, whereas permanent POSH, defined as lasting more than 6 months, is estimated to occur in up to 5% of cases (discussed in (2)). The most recent report on the incidence of POSH is based on analysis of 7852 patients who underwent a total thyroidectomy in Sweden from 2005 to 2015. Permanent hypoPT was defined as treatment with calcium and/or active vitamin D for more than 1 year after surgery and based on this definition, 938 patients (12.5%) developed POSH. In a multivariable analysis, there was a higher risk of POSH among women, patients over the age of 60 years, patients who underwent parathyroid autotransplantation and those operated in centers with a volume of <100 thyroidectomies per year. Surgeons might not be aware of most of these cases as their self-reported data from the quality register only identified 178 of these 938 patients with POSH (4).

Using data from the Danish National Patient Registry, the prevalence of POSH was estimated as 22/100 000 (discussed in (2)). On the background of the constantly increasing number of thyroid operations performed worldwide, the prevalence of POSH is likely to increase in future decades.

## Consequences of postsurgical hypoPT

Recently, the Hypoparathyroidism Patient Experience Scale-Symptom has been developed as a specific tool for patient-reported outcomes in hypoPT (8). One hundred per cent of the concept elicitation patient group experienced physical symptoms attributed to hypoPT, such as tingling, numbness and paresthesia (88%), muscle cramps (86%), physical fatigue (83%), cognitive dysfunction (86%), impaired memory (57%), impaired ability to have a conversation (50%), and a lack of concentration/focus (43%). A total of 17 major signs and symptoms were identified during item generation and these led to the 17-item scoring system (8). The validity of this questionnaire should now be tested in large cohorts.

In our own survey of 252 members of the HypoPara UK (now ParathyroidUK) patient support group, the highest scores for severity of symptoms were 4.8 ± 1.8 for fatigue, and 4.5 ± 1.9 for low sense of well-being. Only 4% of patients never experienced fatigue or numbness/tingling sensations, while 30–35% experienced these symptoms almost all the time. In addition, 149 patients listed a myriad of associated symptoms rarely described in literature (9).

The burden of the disease on individual patients extends to their carers, and this amplifies the impact on health services. In a study of 398 patients with chronic hypoPT not adequately controlled on conventional therapy and 207 caregivers, caregivers’ burden increased with patients’ self-rated symptom severity (10).

Interestingly, the majority of patients are still experiencing symptomatic hypocalcaemia despite regular use of medication and this impacts even more on their quality of life (9). A recent population study of 374 adult patients with hypoPT showed that 75% of sufferers experienced symptoms despite treatment and 79% had required emergency department or hospital admissions (11). This mirrors the results of our survey of HypoPara UK patients, where 93% of responders reported that they required emergency admission to hospital within the last 6 months (9).

In addition to their impaired QoL, patients with hypoPT experience significant long-term morbidity including cataracts, epilepsy and soft tissue calcification. A study of 239 patients with permanent hypoPT identified from the Scandinavian Quality Register for Thyroid, Parathyroid, and Adrenal Surgery found that after a mean follow-up of 4.5 years they had an increased risk for renal insufficiency (HR: 4.88), for any malignancy (HR: 2.15) and for cardiovascular events (HR: 1.88) (12). Using the same registry data it was found that the risk of death was significantly higher among patients with permanent hypoPT after total thyroidectomy (HR: 2.09, 95% CI: 1.04–4.20) (13).

## Current management of post-surgical hypoPT

In the short term, the goals of medical therapy are to reduce symptoms, hospital stay and readmissions, while in the long term, the treatment aims to improve the QoL and reduce the morbidity. Recent European guidelines highlight some of the difficulties in the management of these patients (14). Several medical therapeutic options exist:

1. *Oral calcium and vitamin D supplementation* represent the standard treatment of hypoPT. The most cost-effective approach is to prescribe routine calcium supplements to all patients for 4–6 weeks after bilateral thyroid surgery before reassessing parathyroid function and decide on long-term management. In a meta-analysis of 15 studies with 3037 patients, routine supplementation with calcium + vitamin D3 offered a lower risk of symptomatic (risk difference (RD) −0.25) and biochemical (RD -0.24) hypocalcaemia than treatment based on measurement of calcium levels. The number needed to treat was 4 (95% CI: 3–6) for symptomatic hypocalcaemia (15). This strategy has been labelled as ‘parathyroid splinting’ by researchers in Barcelona, who hypothesised that the recovery of parathyroid function is more likely after a period of intense medical treatment (16).

Historically, most surgeons preferred to monitor calcium levels during in-hospital admission and to select patients who are given calcium supplements based on their symptoms, their slope of calcium changes and/or the dynamic of their PTH levels. Currently, early measurement of PTH in the postoperative period is widely accepted as the best way to identify patients at risk of hypocalcemia following total thyroidectomy (reviewed in (6)). In this way prophylactic calcium and Vitamin D replacement can be initiated early in the postoperative period, reducing the incidence of symptomatic hypocalcemia and avoiding prolonged length of hospital stay.

Daily doses of up to 1200–2000 mg calcium are recommended and this requires regular calcium measurements. Alfacalcidol (1-Alpha-Hydroxycholecalciferol), a vitamin D analogue, is routinely included in the medication of patients with POSH in a dose of 1–3 µg/day. Calcitriol (Rocaltrol®, 1,25(OH)_2_ vitamin D), is the synthetic physiologically-active analogue of vitamin D, and is administered orally at an initial dose of 250 ng daily, and then adjusted up to the usual dose of 0.5–1 µg/day. While Alfacalcidol is used more in the UK, Rocaltrol is used more widely in the USA.

2. *Synthetic PTH* would be the ideal replacement for a hormonal failure, but this medication is not widely available nor routinely used. The drug was accepted for use by the Food and Drug Administration (FDA), USA in 2015 and in the EU in 2017. Both PTH(1-34) or PTH(1-84) improve the bone-remodelling dynamics (17) and the physical and mental functioning (18).

Importantly, PTH(1-34) cannot be discontinued abruptly, as this could lead to severe hypocalcemia and patients have to be slowly weaned over several weeks (19). A further downside of PTH replacement is that it requires daily injections. PTH(1-34) delivered by insulin pump, represents an important advance in the management of hypoPT that has been used in adults and children and proven to achieve a physiological biochemical profile (20, 21).

Two recent developments in this field might make this treatment more ‘user-friendly’. The first is the possibility of delivering teriparatide orally, using nanoemulsion (22). The second is the use of a chemical derivative (TransCon PTH) that is a sustained-release, essentially inactive prodrug transiently bound to an inert carrier, designed to release PTH(1-34). In a phase 1, randomised, placebo-controlled study TransCon PTH administered to healthy adults was generally well tolerated and free PTH demonstrated an effective half-life of approximately 60 h. Based on these data the drug will advance into phase 2 clinical development (23).

The long-term benefits of synthetic PTH have not yet been demonstrated and its safety profile is not fully established. A risk of osteosarcoma was reported in rats but, thus far, skeletal malignancies have not been described by clinical trials in humans (24). Nevertheless, the most recent guidance on FORTEO® (teriparatide) from FDA, USA (August 2013, https://www.fda.gov/media/77492/download) informs healthcare providers of the 2-year maximum lifetime duration of treatment and prohibits its use in children and young adults whose bones are still growing. Furthermore, the FDA Adverse Event Reporting System listed cutaneous calcification and calciphylaxis (https://www.fda.gov/drugs/questions-and-answers-fdas-adverse-event-reporting-system-faers/january-march-2019-potential-signals-serious-risksnew-safety-information-identified-fda-adverse).

## Cost analysis of current medication regimes

In a population-based study from Minnesota, USA the yearly cost of medical care for patients with hypoPT was estimated to be about three times that for healthy patients (2). This however might be a significant underestimate, as the study did not quantify the costs related to utilisation of outpatient clinics, hospitals, emergency departments, or pharmacies, nor the likely costs related to hospitalisation for acute complications of hypoPT.

As a simplified financial model some calculate only the costs related to daily medication. Calcium supplements are cheap and can be bought over the counter. Calchichew (1 gr calcium carbonate two-three times daily for 4 weeks) is approx. £10. Vitamin D supplements (alfacalcidol: 1 µg/day for 1 month) costs under £10. Average retail price for calcitriol (100 capsules, 250 µg) in USA is $35. PTH 1-34 (teriparatide, Forsteo®) is not licensed for use in hypoPT but could be obtained on named patient basis. It is dispensed as a 2.4 mL pre-filled pen (equivalent of 20 µg/day) and it costs £326. PTH 1-84 (Natpara®) is currently used in the ‘BALANCE’ study (https://clinicaltrials.gov/ct2/show/NCT03324880) and is available as multiple-dose cartridge containing different dosage strengths (25–100 µg/dose). The cost of the drug in UK is not available, but the price on the European continent is in the region of £6500 for 28 doses.

## Parathyroid transplantation

As part of routine surgical practice, parathyroid glands that are recognised to have been inadvertently removed are reimplanted during the same procedure into a muscle pocket, with the expectation that they will regain vascular supply and subsequently will maintain their secretory function. The procedure is called autotransplantation, as the patient’s own tissue is transplanted.

In contrast, when discussing other procedures of replacing a deficient organ, such as a kidney transplant or a heart-lung transplant, one refers to using an organ retrieved from another individual (the donor) being reimplanted surgically in a patient (the recipient). The term used to describe this process is allotransplantation. This relies on using a potentially large source of transplantable organs through a rigorous selection process of the donors followed by strict matching with the recipient, based on HLA and blood-groups. The main downside of allotransplantation is the need for life-long immunosuppression medication.

### Parathyroid autotransplantation

One of pioneers of thyroid surgery, Dr Frank Lahey, first performed parathyroid autotransplantation during a thyroidectomy in 1926.

When one (or more) parathyroid gland(s) are inadvertently removed with the thyroid specimen, the need/benefit of autotransplantation is easily accepted. A more difficult scenario is to decide on autotransplantation of a gland deemed devascularised. With increasing surgical experience, one expects to be able to recognise when the blood supply has been compromised and the gland is nonviable, hence the decision of autotransplanting could be justified. More recently, the intraoperative use of autofluorescence with ICG (indocyanine green) has been to assess perfusion of parathyroid glands at the end of the operation. In a study including 196 patients, 11 of the 50 patients in whom no well-perfused parathyroid gland could be identified by angiography, presented with hypoparathyroidism on POD 1, and six on POD 10–15 (7). In their experience, ICG angiography reliably predicts the vascularisation of the parathyroid glands and obviates the need for postoperative measurement of calcium and PTH, and supplementation with calcium in patients with at least one well-perfused parathyroid gland. Whether these findings from the enthusiastic group in Geneva who were early-adopters of this technology could be replicated in other units remains to be determined in future years.

The preferred site for autotransplantation is the sternocleidomastoid muscle. Similar outcomes are expected if re-implantation is performed in the forearm as this has the added benefit of easier monitoring of graft function.

The technique used for autotransplantation is based on personal experience, with a choice between reimplanting 10–20 small slices/fragments of the gland (as described by Wells (25)) or injection of a suspension of parathyroid tissue in buffered saline (26). When performed, it is thought to be associated with a transient hypoPT but a subsequent reduction in rates of permanent hypoPT (26).

## Parathyroid allotransplantation

Recently, parathyroid allotransplantation (PTalloT) has been increasingly discussed as a possible treatment for patients with POSH, after hopes were raised by a case report of a 32-year-old female with intractable hypoPT who underwent successful PTalloT (27).

There are no registered clinical trials involving PTalloT. The topics explored by recent and ongoing trials on hypoPT are summarised in Table 1. Intriguingly, the PARADIGM study from University of California (Pancreatic Islets and Parathyroid Gland Co-transplantation for Treatment of Type 1 Diabetes, ClinicalTrials.gov Identifier: NCT03977662, registered in 2019), aims to assess the effectiveness of co-transplantation of allogeneic parathyroid cells with adult pancreatic islets. The hypothesis explored is that the parathyroid glands, a richly vascularised organ that can be transplanted routinely in the muscle, can provide the CD34+ vascular endothelial progenitor cells that reside within PTG, and such cells will induce neoangiogenesis and promote islet engraftment and survival when co-transplanted with the pancreatic islets.

**Table 1:** Topics addressed by studies on hypoparathyroidism identified on *clinicaltrials.gov* registry (accessed on 1 May 2020).

Topic	Number of studies
Epidemiology of hypoPT	5
Use of synthetic PTH as treatment for hypoPT	26
Techniques for reducing the incidence of post-thyroidectomy hypoPT	14
Care of patients with genetic syndromes associated with hypoPT	30

Several factors have to be considered when planning PTalloT:

### 1. Selection of donors of parathyroid tissue

- *Living donors*: A case report from the Regensburg, Germany, described a 32-year-old female who underwent a living-donor allotransplantation of two healthy parathyroid glands from her brother (27). Similarly, parathyroid tissue was retrieved as a simultaneous additional surgical procedure during donor nephrectomy and transplanted into the same patient (28, 29).
- *Brain-dead donors*: A single case report from Antwerp, Belgium, described a woman with terminal renal failure and a previous total parathyroidectomy who underwent combined kidney/parathyroid transplantation from a brain-dead donor (30).
- *Hyperplastic gland(s) from patients with secondary hyperparathyroidism* is the most common source of viable parathyroid tissue for transplantation reported to date. Because multigland hyperplasia is a physiological response to the biochemical abnormalities triggered by chronic renal failure, it is postulated that such glands should return to normal function once successfully transplanted in a patient with normal renal function.
- *Adenomatous glands from patients with primary hyperparathyroidism (PHPT)*: The first case report was published over three decades ago (31). This option is attractive, because of the large number of potential donors. Many patients with PHPT have minimally raised calcium levels and their set-point for the calcium-PTH curve is only minimally shifted to the right by the monoclonal expansion of parathyroid cells with abnormal expression of the calcium-sensing receptor (CaSR). Such cells could represent a reasonable source of cells if the donor had an adenoma with a setpoint close to upper range of normal calcium levels. Reassuringly, xenografts from parathyroid adenomas into rabbits showed no histological changes during follow-up without any reported malignant transformation (32) but such data is yet to be published for allografts.
  This approach raises concerns that a successful allotransplantation could induce hyperparathyroidism (and its associated morbidity) in a patient previously suffering with hypoparathyroidism. This has not been reported and the risk could be mitigated by using parathyroid adenomas with a calcium threshold close to normal (i.e. donors with calcium levels just above normal range). Furthermore, if hyperparathyroidism becomes more distressing than the initial hypoparathyroidism, patients could have the graft removed.
- *Parathyroid-like cells derived from human embryonic stem cell lines* would be optimal for cellular replacement, as each cell contains the complete functions of the organ, and parathyroid cells do not have requirement for an architectural structure for their function (33). Future progress in this field could make allotransplantation a very attractive option but at this point such cells are not available.

### 2. Parathyroid cell preparation before allotransplantation

Critical to preserving parathyroid function after PTalloT is ensuring a reasonable cell yield and maintaining the viability of the cells. Several solutions have been explored.

*University Of Wisconsin solution* is commonly used. For example, some reported successful transplantation with cold ischemia time of 14 h (30).

*Parathyroid transport solution* reported from Bezmialem Vakif University, Istanbul, Turkey, was designed specifically for parathyroid tissue transplantation. This has a pending patent and is reported to support cellular viability and PTH release and to protect CaSR functionality for up to 24 h of cold ischaemia (34).

*Microencapsulation of parathyroid tissue* is a technique based on protecting the tissue to be transplanted from the immunologic response by coating it with a semipermeable membrane. This could allow allotransplantation without immunosuppression (35).

In a report from the University of Chile, commercial sodium alginate was used for microencapsulation and the graft functioned for at least 20 months (36). At the Bezmialem Vakif University, Istanbul, Turkey, microencapsulation of cultured parathyroid cells was performed with ultrapure alginate and spheroids were generated with calcium chloride. Microencapsulated parathyroid cells showed the functionality for more than 1 year (37). At the University of Wurzburg, Germany, tissue particles and single cells/cell clusters were microencapsulated with amitogenic Ba(2+) alginate. Preoperative evaluation of microencapsulated parathyroid tissue revealed differences in function and thus facilitated selection of the optimal bioartifical graft for human PTalloT (38).

*Thermoreversible gel* made of polyethyleneglycol-polyalanine-co-phenylalanine (PEG-PAF) (sol form at 4°C, gel form at 37°C) was manufactured by researchers at the Hallym University, Chuncheon, Korea. On day 70, the PTH level was restored by just over 50% and histology confirmed the successful transplantation of parathyroid tissues (39).

*Macrocapsule* containing 20–30 × 10^6^ parathyroid cells was constructed by researchers in Minsk, Belarus using polyvinylidine difluoride and implanted into the deep femoral artery followed by at least 3 months of functional activity (40). No other group has replicated this technique.

### 3. Site of transplantation

The commonly used method is injection into the left deltoid muscles (41) and forearm muscles (27), but some centres used laparoscopic transplantation in the omental tissue (37). In animal models, the thymus (42) and the adrenal glands (43) appear to be a privileged sites for PTalloT, but these experimental data would have limited clinical applicability.

### 4. Timing of transplantation

The initial paper, published in 1996 from the Medical Academy, Warsaw, Poland, described allotransplantation after culturing of cells (44). In that report, 85 patients underwent 116 allotransplantations of cultured parathyroid cells. After 6 weeks of cultivation and freezing, the parathyroid cells decreased their normal HLA (HLA) class I ABC expression and were free of HLA class II positive cells. The viability of cultured cells was 95%. In 64 patients the allografts retained their endocrine function for more than 2 months (45).

In a more recent report, the parathyroid glands resected from the living donors with secondary hyperparathyroidism were fragmented quickly in the operation room before being injected into the left deltoid muscles of the two recipients (41).

### 5. Impact of HLA typing on success of parathyroid allotransplantation

Surprisingly, HLA-matching might not be significant for successful PTalloT. Only one study evaluated the role of extensive immunological tests before PTalloT. Four unrelated recipients whose grafts functioned 1–4 years and one living donor were evaluated for HLA-typing, PRA (panel reactive antibodies), and CXM tests (compatibility test Xmatch). Two out four patients were negative for *de novo* donor specific antibodies and three out of four of the recipients remained negative for PRA (37).

### 6. Use of immunosuppression after parathyroid allotransplantation

Some centres have not used immunosuppression after PTalloT (46). Metylprednisolone as a short course (2 days postop) has been used in two successful allotransplants with a rapid return to normal calcium levels within 1 month and lasting over 1 year (41, 47). Similarly, in another report methylprednisolone was used for 1 month after allotransplantation (48).

Tacrolimus, mycophenolate mofetil and steroids were used by a group in Cleveland, USA, for allotransplantation of cryopreserved parathyroid tissue in a kidney transplant recipient (49). When an immunosuppressive regimen of 6 months was used by researchers from Guadalajara, México, none of the patients had immunosuppression side effects and four out of five grafts maintained PTH secretion for 2 years (50).

### 7. Expected long-term outcomes after parathyroid allotransplantation

The longest survival reported to date is from the University Hospital Regensburg, Germany, where a 32-year-old female had a living-donor allotransplantation and remains with normal calcium/PTH levels 3 years later (27).

A recent systematic literature review summarised the evidence from five studies and reported a mean allograft survival of 47% (95% CI 24–71%) when patients were followed-up for < 6 months and 41% (2.3–80%) when followed-up for up to 1-year. In further five studies, patients were followed up > 12 months and the mean graft survival was 46% (95% CI 11–80%) (51).

### 8. Symptomatic benefits after allotransplantation

The recent systematic review found that only two studies reported the impact on symptoms related to POSH (51). Chvostek and Trousseau’s signs disappeared in over 90% of patients and most could carry out basic daily activities without having signs of hypocalcaemia, which was not possible before transplantation (51).

## Final comments

Permanent hypoparathyroidism is currently the most common and debilitating complication of thyroid surgery. In parallel with efforts to improve its medical management, there is growing pressure to reduce its incidence by ensuring such operations are done in high volume units. In 2019, the European Society of Endocrine Surgeons reviewed the evidence for volume-outcome correlation in thyroid surgery and reported that the incidence of complications (recurrent laryngeal nerve injury and hypocalcaemia/hypoparathyroidism) is lower when the surgeon performs more than 30 operations yearly. The ESES recommends therefore that routine thyroid cases should be operated in centres performing at least 25/year and surgery for thyroid cancer should be centralised in units performing at least 50 thyroid operations/year (52). Similar recommendations are expected soon in the United Kingdom after completion of a national review of service delivery for endocrine surgery under the acronym GIRFT (*get it right first time*).

The options available for patients with severely symptomatic hypoPT are summarised in Table 2. Though the clinical need for a new effective treatment in patients with poorly controlled hypoPT is very high, data available so far regarding long-term outcome of allotransplantation are deemed as unconvincing and therefore PTalloT has remained restricted to a very small number of units.

**Table 2:** Comparisons of current medical therapy for permanent hypoparathyroidism.

	Advantages	Disadvantages
Oral calcium and vitamin D supplements	Cheap, easily available	Need for regular blood tests
	Safe	Ineffective in many patients
Synthetic PTH	Ideal hormonal replacement	Expensive
	Very effective when administered with a pump	Long term unknown
		Not suitable for needle-phobic patients
		Restricted use up to 2 years due to concerns regarding development of osteosarcoma
Autotransplantation	Easy to perform	Need for cryopreservation facilities
	No need for immunosuppression	Unpredictable survival or preserved tissue
Allotransplantation	Potentially unlimited source of donor cells	Alleged need for immunosuppression
	Could be repeated	Cost of HLA matching and the surgical procedure
		Need for graft monitoring

Patients’ expectations remain disproportionate compared with the enthusiasm of clinicians to consider this as a viable therapeutic option. Is likely that a mean transplant survival of 12 months is unlikely to be a compelling prospect for patients. There is, therefore, a need to define better the benefits and the limitations of PTalloT.

At this point, PTalloT should be offered only for patients who remain severely symptomatic despite intense medical therapy. This approach can only be assessed in a prospective multicenter study that should build on the experience of single centers. Until its effectiveness is convincingly demonstrated in a study with 3–5 years follow-up, PTalloT will remain as an experimental procedure restricted to a small number of units who showed enthusiasm with the technique.

Based on the data available, our view is that the source of parathyroid cells should be adenomas from patients with mild hyperparathyroidism or hyperplastic glands from patients with secondary hyperparathyroidism because the pool of donors would be large enough. Cells from the resected parathyroid adenomas could be cultured and assessed in the laboratory for viability and function for a short period of 1–2 days, before transplantation into a forearm muscle pocket.

The need and extent of immune suppression should be explored prospectively in a randomised fashion. It is encouraging that some reported successful allotransplantation without long-term immunosuppression as it remains likely that daily medication with standard immunosupressants (tacrolimus, everolimus) or even the new co-stimulation blocker iscalimab (Novartis) might trigger side effects that could outweigh the benefits of avoiding/reducing the use of calcium-vitamin D supplements.

**Figure 1:**
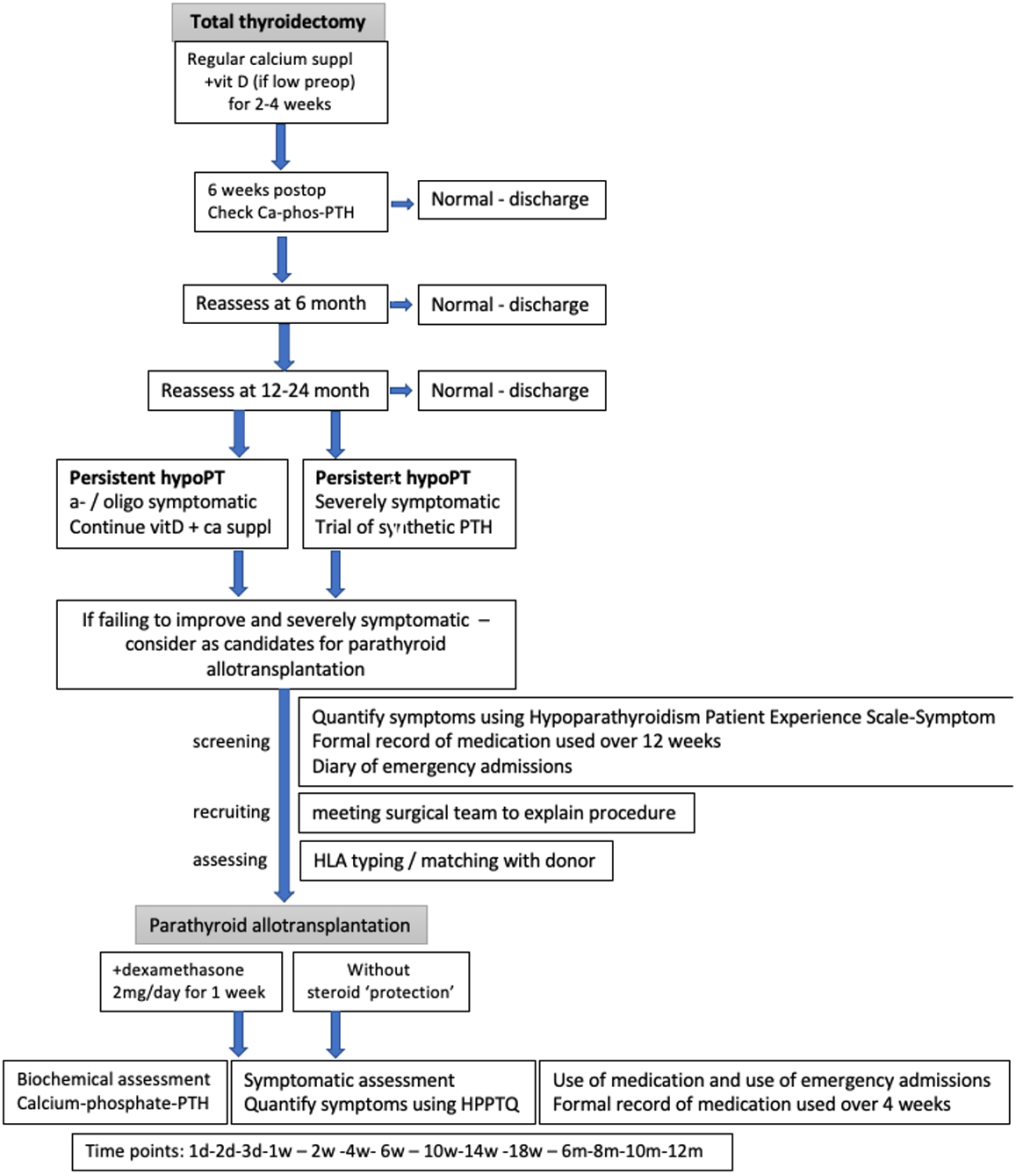
Proposed strategy for recruitment into a prospective trial of parathyroid allotransplantation for patients with severe hypoparathyroidism.

**Figure 2:**
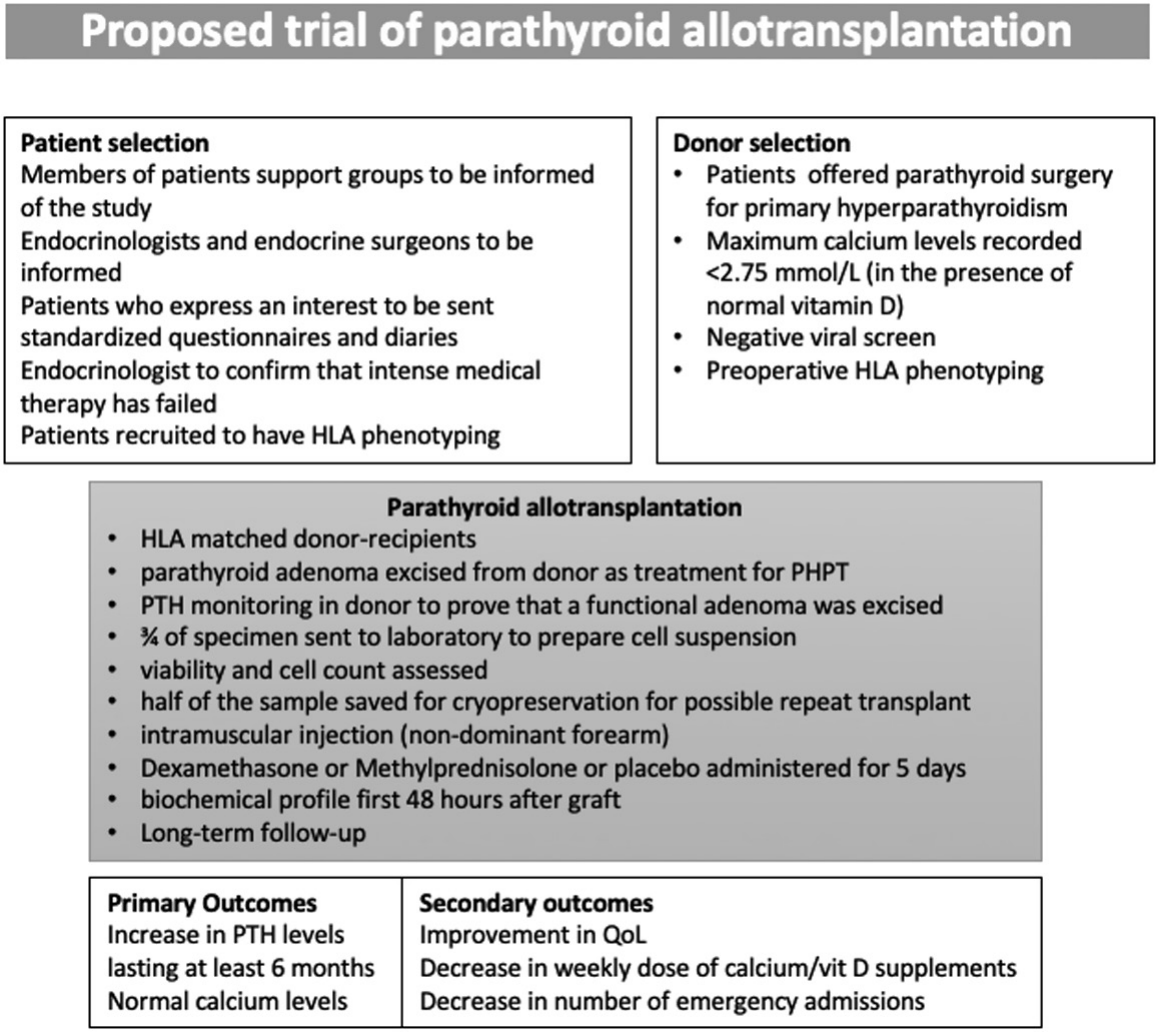
Proposed design for a randomised trial of parathyroid allotransplantation.

Recruitment of patients into such a trial and the proposed design of such a trial are outlined in Figs 1 and 2, respectively. In our recent paper investigating the views of members of HypoPara UK group, at least 40% of 252 respondents expressed an interest participating in a clinical trial that would explore the feasibility and benefits of PTalloT (9). In addition to analysing biochemical parameters, such a study should determine if the improvement in quality of life becomes apparent and is sustainable.

A prospective cohort trial, rather than more case reports, should be the next step in this process and such collaboration between endocrinologists and thyroid surgeons could make a significant impact on the care of a group of patients with a debilitating chronic condition.

## Declaration of interest

The authors declare that there is no conflict of interest that could be perceived as prejudicing the impartiality of this review.

## Funding

This work was supported by the National Institute for Health Research (NIHR) Oxford Biomedical Research Centre (BRC) Programme (to R V T).

## Author contribution statement

Both authors have contributed to the planning and writing of this review.
